# Allogeneic Stem Cell Transplantation: The Relevance of Conditioning Regime Intensity for Myelodysplastic Syndromes (MDS)

**DOI:** 10.3390/curroncol32060319

**Published:** 2025-05-30

**Authors:** Tobias Berg, Brittany Salter, Michael Radford, He Tian Tony Chen, Brian Leber

**Affiliations:** 1 Department of Oncology, McMaster University, Hamilton, ON L8V 5C2, Canada; bergt1@mcmaster.ca (T.B.); radfordj@hhsc.ca (M.R.); 2 Department of Medicine, Division of Hematology, McMaster University, Hamilton, ON L8V 5C2, Canada; brittany.salter@medportal.ca; 3 Faculty of Medicine, McMaster University, Hamilton, ON L8S 4L8, Canada; chenht@mcmaster.ca

**Keywords:** transplant, myelodysplastic syndrome, conditioning regime

## Abstract

Allogeneic hematopoietic cell transplantation (alloHCT) is the sole curative therapy for myelodysplastic syndrome (MDS). While alloHCT clearly confers a significant survival advantage in high-risk MDS, it is less clear how the disease burden and impact of conditioning intensity impact survival. This review addresses critical issues surrounding this topic, emphasizing the unique cell biology of MDS and the evolving concepts of conditioning intensity compared to other diseases, including acute myeloid leukemia (AML). The review is structured around three interconnected themes. First, it clarifies the varying interpretations of conditioning intensity. Second, it examines the interplay between disease burden at transplant and conditioning intensity in determining outcomes, including a comparative analysis with acute myeloid leukemia (AML) to highlight similarities and differences. Third, it explores the relationship between conditioning regimen intensity and immune reconstitution, particularly focusing on the graft-versus-tumor (GvT) effect and its potential modulation by conditioning intensity. Understanding the stem cell target of conditioning regimens is emphasized, as the persistence of the underlying MDS stem cell necessitates a thorough understanding of this concept for improved therapeutic strategies.

## 1. Introduction

Allogeneic hematopoietic cell transplantation (alloHCT) remains the only curative therapy for myelodysplastic syndrome (MDS) [1]. In the large Blood and Marrow Transplant Clinical Trial Network (BMT CTN) 1102 study, alloHCT demonstrated a survival advantage in high-risk MDS [2]. The role of conditioning regimen intensity and the type of alloHCT utilized for patients with MDS remains a contentious topic, having been the focus of extensive discourse over the past several decades. Several critical issues must be addressed when examining this topic, including an understanding of the unique cell biology of MDS and the evolving concepts of conditioning intensity that have been applied to other diseases.

This review will be organized into three interrelated themes. The first theme explores the concept of intensity, highlighting the various interpretations that can lead to confusion when referencing different levels of this concept. The second theme examines whether the amount of disease present at the time of transplant is a significant factor in determining outcomes and how conditioning regime intensity interacts with this variable. A comparative analysis will be conducted between MDS and acute myeloid leukemia (AML), where the results are more definitive. Though not identical, the similarities between MDS and AML will provide insights and clarify some of the complexities involved. The third theme addresses the interplay between conditioning regime intensity and immune reconstitution, including the graft-versus-tumor (GvT) effect. This relationship has been investigated in randomized trials, suggesting that conditioning intensity may influence the onset and efficacy of the GvT effect.

## 2. The Concepts of Intensity and Its Stem Cell Target, Biologic Bases, and Pragmatic Considerations

The target of the conditioning regimen, regardless of intensity, is the stem cell that gives rise to the MDS or leukemia. It is exceedingly rare for the bulk of the MDS disease to persist through the conditioning regimen. The concerning presence of relapse in this population is likely due to the underlying stem cell that has not been eradicated by the drugs in the conditioning regimen, unlike its more differentiated progeny. Therefore, it is essential to examine the most current understanding of the stem cell concept.

The original concept of a leukemic or MDS stem cell was that of a rare cell with an incompletely defined immunophenotype, assayed in immunocompromised mouse model systems, possessing properties of self-renewal as well as differentiation. Many intrinsic properties of the cell, such as quiescence with the attendant metabolic state and protection from intracellular toxins by exporter pumps, contribute to its relative resistance to standard chemotherapy agents and are therefore thought to be major contributors to the pool of cells that generate relapse. This mirrors the differentiation pathway of a normal stem cell, with leukemia conceptualized as a truncation of this branching pattern in distinct ways. More recently, this concept of a tree diagram with the stem cell at the top of a simple branching hierarchy leading to the different end-stage cells beneath it has been expanded and deepened. By examining the various transcriptional states on a single-cell basis that leukemic cells assume along the “normal” pathways of differentiation [3] from the pluripotent stem cell to cells with more defined lineage commitments, a groundbreaking study [4] indicated that the leukemic stem cell of a patient could be positioned after deconvolution of the transcriptome of the bulk leukemic population to a point within this complex landscape; for analytical purposes, this landscape can be conveniently collapsed into two main dimensions. Further analysis of this concept demonstrated that this could provide an excellent bridge to the current understanding of the genetics of leukemia, as specific mutational types or chromosomal translocations (e.g., *NPM1*-mutated AML or core-binding factor-rearranged AML) could be situated within this specific landscape [4]. This and related work [5] illustrated the intriguing concept that after treatment, the location of the leukemic stem cell detected at relapse could shift to an adjacent point in this multi-dimensional landscape, accompanied by a concomitant change in metabolic state, immunophenotype, etc. Thus, this represents an elegant and precise method of detecting the changes wrought by intervening therapy. As complex as this process is, further technical refinement [6] has expanded this from “one point” on this landscape for the putative stem cell from a patient to reveal what is essentially a smear on this landscape when single-cell multiomics from the leukemia cells of a patient are analyzed as populations of single cells. For practical purposes, this implies that the stem cell “pool” that initiated the disease could differ substantially in its metabolic properties from the pool present at the time of transplant if therapy has intervened in the interim [7]. The concept of leukemia or MDS being a village of clones only begins to capture the complexity of the situation and the target that must be eliminated: it is more challenging to eradicate a village of criminals with different and unstable attributes than a lone miscreant.

The other aspect of the tremendous technical and conceptual progress in understanding the genetic and biological basis of MDS and AML also has implications within this framework. It is well recognized within the most recent proposals [8,9] for the pathological diagnosis of MDS and AML that these diseases exist on a biological and clinical continuum. There is a high degree of consistency between the two recently published schemes, although some discordance is pragmatically bothersome in the differences in nomenclature used [10]. Nevertheless, there is broad agreement on a continuum along one or a few dimensions in the MDS-AML “spectrum.” Within MDS and AML, distinct entities are recognized by their genetic and/or karyotypic abnormalities in both classification systems. The intimate relationship between MDS and AML is underscored by the fact that many of these mutations are common to both diseases. However, the absolute practical necessity of placing a patient pathology sample somewhere along the spectrum for diagnostic and therapeutic decisions likely underestimates the complexity and interrelation of mutations between cases, especially in what can broadly be termed an overlap category. A recent informative analysis of these data [11] by an unbiased machine learning approach has indicated that rather than being distinct entities defined by one or a few genes or karyotypic abnormalities, there appear to be 14 distinct clusters of diseases defined by a combination of co-occurring mutations and/or chromosomal abnormalities along the high-grade MDS-oligoblastic AML spectrum. These data allowed the investigators to demonstrate the prognostic implications of this clustering for patients exposed to standard chemotherapy, but the prognostic significance of this with respect to susceptibility to intermediate- or high-dose chemotherapy in transplant conditioning regimens remains unanswered and is an important area for future investigation. Will this grouping also correspond to discrete entities on the multi-dimensional transcriptome stem cell landscape described above? Will this grouping, which is a priori and unbiased, represent a better method of “carving nature at its joints” and be the most relevant way to determine prognostic grouping for transplant sensitivity? Many of these questions remain unanswered.

The complexities surrounding the nature of leukemic stem cells and the continuum between MDS and AML provide essential context for the pragmatic interpretation of clinical studies we will review. Understanding these multifaceted dimensions will be key to accurately assessing treatment outcomes and tailoring therapeutic strategies in the future. As research continues to evolve, these considerations will serve as a foundation for future investigations to improve outcomes for patients undergoing a transplant for MDS.

The concept of conditioning regimen intensity is intricate when applied to clinical data, as it encompasses multiple distinct dimensions. The initial distinction recognizes that the original conditioning regimens developed for acute leukemia were subsequently adapted for various age groups that could not tolerate standard high-intensity treatments. This adaptation has expanded the population of patients eligible for transplants. Over several decades, we have transitioned from therapies only applicable to children and fit young adults into those that can now be offered to all age groups. Consequently, discussions surrounding treatment intensity have included both the myeloablative and immunosuppressive effects of diverse regimens. Each aspect is significant for distinct reasons: the former serves as a surrogate for disease control through chemotherapy or radiotherapy, as elaborated upon in subsequent sections, while the latter aims to suppress the endogenous immune system to facilitate engraftment by avoiding rejection and allowing establishment of the donor’s immune system in the recipient, which is essential for achieving the GvT effect. This distinction has gained widespread acceptance in review articles, textbooks, and guidelines issued by transplant societies [12], often illustrated within a two-dimensional axis that represents relative intensity across myeloid and lymphoid compartments. Nevertheless, the precise positioning of a regimen along these axes frequently relies on an expert clinical consensus rather than a comprehensive understanding of the pharmacology of the drug combinations. A second aspect involves grading regimens based on their non-hematopoietic side effects, particularly those affecting rapidly dividing cell populations, such as the gastrointestinal tract, other drug-specific organ toxicities, and hematopoietic toxicity (whether stem cell support was “required”). The widely utilized scale of conditioning regimen intensities published by the European Society for Blood and Marrow Transplantation (EBMT) effectively integrates intensity with the impact on these cell populations [13,14].

**Figure 1:**
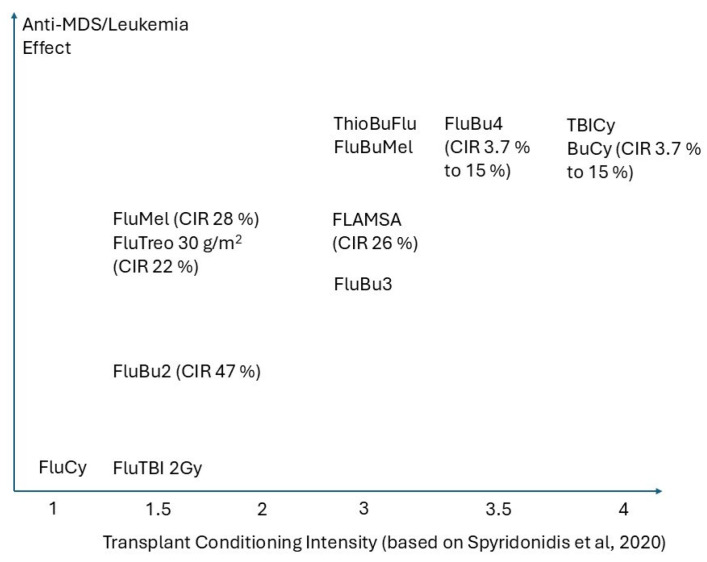
Transplant Conditioning Intensity (based on Spyridonis et al., 2020 [13]).

A so far under-recognized third aspect, likely most relevant for disease control, pertains to the intensity of the anti-MDS effect of each regimen on the malignant clone. This is closely related to, but not synonymous with, its myeloablative effects on normal hematopoiesis or intensity in terms of organ toxicities. Unlike AML, where myeloablative conditioning (MAC) has generally proven to be superior to reduced intensity conditioning (RIC) in fit individuals, prospective studies in MDS have thus far shown no survival advantage to more aggressive conditioning. The BMT CTN 9091 RCT compared MAC (Flu/Bu4 and Bu/Cy) vs. RIC (FluBu2, Flu/Mel) in a cohort of AML and MDS patients, finding an overall survival (OS) advantage for MAC in the AML subgroup (n = 218), but not the MDS subgroup (n = 54) [15]. An initial increase in relapse incidence was seen in the MDS RIC group, primarily driven by relapses before 12 months, but it did not continue to be significant at the 4-year follow-up analysis, which also showed no OS difference between regimens [16]. The larger MDS cohort in the RICMAC RCT (n = 127) similarly showed no clear advantage between MAC (Bu/Cy) and RIC (Flu/Bu2), with a borderline advantage for RIC likely attributable to the use of the more toxic Bu/Cy as the MAC comparator rather than Flu/Bu4 [17]. Interestingly, this dataset also shows a transient pattern of increased relapse incidence associated with RIC limited to the pre-12-month timepoint [18]. Given that the slowest dividing hematopoietic stem cell populations have cycling times upwards of 6 months, the choice of conditioning may have a less consequential impact on more quiescent hematopoietic stem cell populations responsible for delayed relapse, leading to a lack of outcome difference despite an apparent suppression of early relapse [19]. A proposed scheme that depicts the anti-leukemic/MDS effects of various conditioning regimes compared to conditioning intensity is presented in Figure 1.

The FIGARO trial by the British transplant group investigated the impact of sequential intensified conditioning (FLAMSA-RIC) versus RIC on AML/MDS transplant outcomes (n = 164/80) [20]. The findings were predominantly negative regarding the primary outcome, again suggesting that conditioning intensification does not yield a strong difference in outcomes in either the AML or in MDS subgroups. However, in the context of these negative findings, it is worthwhile noting some of the details of the intensification used and the comparator. First, 31 out of 88 patients assigned to the RIC arm received fludarabine and melphalan, which, as further discussed below, has a significantly higher anti-leukemic activity. Second, in the dose intensification arm, for patients >60 years, the targeted busulfan dose of 8.4 mg/kg had to be reduced in the latter 2/3 of the cohort to 6.4 mg/kg, the dosing schedule in the control arm. Thus, for most patients older than 60, the dose intensification did not include the alkylating agent but rather the amsacrine (100 mg/m^2^ × 4) and intermediate-dose cytarabine (1 gm/m^2^ × 4), both agents that, while being quite effective in inducing a remission in the setting of acute leukemia, probably have minimal impact on quiescent leukemic stem cells. These two factors may have attenuated the planned dose intensity differences between the control and experimental arms.

As a follow-up to the FIGARO trial, the UK IMPACT COSI trial (NCT04217278) will evaluate the effect of a protocol containing two alkylating agents, thiotepa and busulfan, combined with fludarabine (TBF), which in retrospective analyses has been shown to exhibit a reduced relapse rate in AML [21]. Recent studies suggest that agents within the same class exhibit varying dose–response curves in terms of both anti-leukemic effect and toxicity. While comparisons between MAC and RIC protocols in MDS have shown no significant clinical differences, there are notable distinctions between alkylating agent-based regimens with similar dose intensities. In a large retrospective CIBMTR analysis, Oran et al. compared RIC protocols for older MDS patients and found that fludarabine–melphalan conditioning resulted in lower relapse incidence and superior disease-free survival at both 1 and 3 years compared to fludarabine–busulfan, alongside improved OS at 3 years, despite increased treatment-related mortality (TRM) [22]. The potent anti-leukemic effect of melphalan was first observed in relapsed AML patients treated with a salvage regimen followed by autologous stem cell re-infusion, which achieved a high second complete remission (CR) rate of 93%. This success led to the development of melphalan-based sequential conditioning regimens [23], which contributed to the success of the ASAP trial in refractory AML patients [24]. However, recent data from the EBMT suggest that sequential conditioning does not improve survival outcomes in higher-risk MDS patients with excess blasts undergoing alloHCT, where prognosis is primarily influenced by baseline disease risk and patient factors [25].

A promising advance in conditioning could be using the alkylating agent treosulfan. In a phase 3 trial comparing conditioning regimens for older or comorbid AML or MDS patients undergoing allo-HCT, treosulfan combined with fludarabine demonstrated superior 2-year event-free survival (64.0% vs. 50.4%) compared to busulfan plus fludarabine, with a comparable safety profile [26]. A retrospective EBMT analysis supported these findings, focused on MDS patients [27], and another cohort study, where the introduction of treosulfan as a preferred conditioning regimen for MDS patients resulted in improved survival outcomes [28]. A selected summary of the results of trials and analyses of conditioning regimes is presented in Table 1.

## 3. The Impact of Disease Burden and Cytoreduction on Patient Outcomes in Patients Who Receive Allogeneic Hematopoietic Stem Cell Transplant for MDS

In 2023, the International Working Group (IWG) published updated response criteria to assess treatment responses for patients with MDS. However, this criterion has not yet been validated to predict outcomes for patients undergoing allo-HCT. Existing prognostic scores, including IPSS, IPSS-R, and IPSS-M, are used to predict outcomes, including OS and leukemic transformation, at the time of MDS diagnosis, but these prognostic scores are less reliable when predicting outcomes following allo-HCT [32]. There have been several scoring tools developed to predict post-allo-HCT outcomes in MDS, including a CIBMTR transplant-specific scoring system [33], transplantation risk index [34], and EBMT transplant-specific risk score [35]. The most recent scoring system also integrates optimal time to transplant based on age and IPSS-M as a relevant variable to predict outcome [36].

These scores include age, performance status, peripheral blood blasts, cytogenetics, platelet count, HCT-CI score, and refractoriness to chemotherapy. Although these scores include several important patient factors, they do not fully encapsulate the disease burden at the time of allo-HCT and how this may influence long-term outcomes. This has been an ongoing concern for more than two decades, as it has been clear that the question of whether cytoreduction can optimize the disease burden in higher-risk MDS patients while waiting for allo-HCT needs to be addressed. However, analysis of the data at that time indicated that the use of cytoreductive therapy to obtain CR prior to allo-HCT did not appear to confer benefit, and consideration was given to the potential interference with transplant receipt performance status if the donor was readily available. If cytoreduction was to be used, when weighing complications related to intensive chemotherapy, there appeared to be a benefit in post-allo-HCT outcomes when compared to HMA for cytoreduction in MDS [29]. Since this original assessment, despite further research, the problem remains vexing.

Achieving a CR before allo-HCT is not a firm requirement in MDS. There is evidence to suggest that disease burden prior to allo-HCT impacts long-term patient outcomes [36], but whether or not to attempt cytoreduction before allo-HCT still remains controversial. Many studies have shown that the percentage of bone marrow (BM) blasts at the time of transplantation significantly influences outcomes after allo-HCT in MDS, with a higher risk of relapse and worse OS [37,38,39,40,41,42,43,44]. Patients who have achieved CR before allo-HCT have been shown to have better relapse-free survival (RFS) and OS following transplantation in numerous publications [37,39,40,42,45,46]. However, a notable limitation of these studies was the assessment of BM blast count among patients treated with cytoreductive therapy and the exclusion of patients who received upfront allo-HCT. As such, these studies have an inherent selection bias for patients with arguably higher-risk diseases who are chemotherapy-refractory and, unsurprisingly, do worse following allo-HCT. In 2019, Schroeder et al. looked specifically at BM blast count in patients who received upfront allo-HCT and found no difference in RFS or OS in those with BM blast count ≥10% or <10% [47]. A follow-up study in 2024 reported outcomes of 109 MDS patients who underwent allo-HCT with or without HMA pre-treatment, with 36.7% of those having 10–19% blasts and 50% having intermediate or higher-risk cytogenetics, with 65% of patients in the upfront allo-HCT group and 34% in the HMA-based group [47,48]. Of those who received HMA therapy, only 39.4% achieved CR before allo-HCT, resulting in a comparable blast count grouping to the upfront group at the time of transplant. At a median follow-up of 63 months, the 5-year OS and RFS were lower in the HMA versus the up-front group, at 37.4% versus 65.8% and 30.95% versus 53.4%, respectively. In the multivariate analysis, blast count did not impact OS, RFS, or relapse incidence. In addition, of the 34% who achieved CR after HMA, there were no superior outcomes when compared to upfront allo-HCT. In a large recent Nordic MDS study investigating the role of MRD monitoring post-transplant, there was no effect of blast count pre-transplant on RFS or OS [48]. These findings are supported by Kounma et al., who reported that intensive induction chemotherapy prior to allo-HCT did not reduce relapse or increase survival compared to upfront allo-HCT [49]. In addition, Chen et al. reported that upfront allo-HCT achieved similar outcomes compared to patients who received pre-transplant azacitidine [50]. A large retrospective study demonstrated that higher-risk MDS patients or those with poor-risk cytogenetics had inferior outcomes when transplanted in CR following intensive chemotherapy compared to those who received upfront allo-HCT [51]. More recent studies have demonstrated that in patients treated with HMA, achieving a CR prior to allo-HCT does not provide additional OS benefit when adjusted for baseline IPSS-M risk [52]. Using a more global indicator of treatment response, an analysis of a large EBMT database indicated that “successful” pre-transplant therapy with HMA as measured by downstaging of the IPSS-R stage (diagnostic marrow compared to pre-transplant assessment) showed no improvement in OS, PFS, or cumulative incidence of relapse [53,54]. By contrast, there was a decrease in relapse incidence and improvement in PFS in patients with improvements in IPSS-R score after intensive chemotherapy, but this was a non-randomized comparison, and this latter group was significantly younger with shorter disease duration pre-transplant compared to patients treated with HMA.

Current guidelines recommend upfront allo-HCT in higher-risk patients with a lower disease burden (<10% bone marrow blasts); however, for patients with higher disease burden (≥10% bone marrow blasts), cytoreduction could be considered, especially in patients with RIC [55,56]. There are no clear recommendations on whether induction should entail intensive chemotherapy versus non-intensive therapies such as HMA [57]. Considering the recent findings of Schroeder et al. and the EBMT database analysis, this does challenge the current practice for the higher-risk MDS or MDS/AML patient subsets and reinforces that upfront allo-HCT is a feasible and potentially superior strategy compared to cytoreduction prior to allo-HCT, especially in those patients deemed unfit for standard induction chemotherapy.

Unfortunately, at present, there are no randomized prospective studies that have compared intensive versus non-intensive cytoreduction prior to allo-HCT in patients with MDS to give a firm grounding to the above suggestion. If a clinician decides to proceed with cytoreduction, it is unclear what the most efficacious strategy would be (i.e., intensive versus non-intensive). The multinational VERONA clinical trial (NCT04401748) has completed accrual and has compared HMA monotherapy with HMA + venetoclax in newly diagnosed MDS patients; the highly anticipated results will let us know if there is a safe way to improve disease control and deepen response. Transplant is not part of the trial design; however, given the precedent of the widespread use of the combination of azacitidine and venetoclax as a bridge to transplant in AML, it is anticipated that there may be rapid uptake of this strategy in MDS if the trial shows positive results. For patients fit for induction chemotherapy, the PALOMA trial (NCT04061239) compares CPX-351 versus standard of care (either hypomethylating agent or 7 + 3) before transplant as part of the study design. The details of the transplant protocols are not specified and are deferred to institutional standards. As interesting as the findings of these two trials will be, one key limitation is that they do not include a comparative upfront allo-HCT group.

There have been many other non-randomized retrospective studies that have looked at different cytoreduction regimens in MDS prior to allo-HCT. These have shown that intensive chemotherapy and HMA have comparable survival and relapse rates but better tolerability with HMA [58,59]. The use of intensive chemotherapy is associated with toxicity and TRM in up to 16% of patients with MDS [43]. However, HMA therapy is not without its own toxicities, and there is a risk of prolonged myelosuppression, infection, and organ toxicity, particularly in frail or elderly patients [60,61]. A systematic review and meta-analysis reported findings from 18 studies where there was no difference in OS (HR 0.92), risk of relapse (HR 1.08), or non-relapse mortality (NRM) (HR 0.93) when comparing upfront transplantation versus cytoreductive therapy [37]. However, achieving CR prior to allo-HCT was associated with a non-significant improvement in RFS (HR 0.80, *p* = 0.054) and decreased (HR 0.53; *p* = 0.018) when compared to upfront transplantation. Lastly, when comparing induction regimens, there was no difference in outcomes for HMA versus intensive chemotherapy. Based on these findings, they suggested that timely allo-HCT should be considered in patients with high-risk MDS and that cytoreductive therapy could be considered during donor search to reduce disease burden prior to allo-HCT to improve long-term outcomes. Clear results of the aforementioned randomized trials will likely provide critical information about making these difficult decisions.

## 4. Relationship Between Somatic Mutations and Conditioning Intensity for Allogeneic Hematopoietic Stem Cell Transplant in MDS

Molecular abnormalities have been identified in more than 90% of patients with MDS and represent important markers of disease prognosis. The newer IPSS-M score incorporates somatic mutations to help prognosticate patients at diagnosis; as mentioned, the most recent proposed prognostic scoring tools incorporate somatic mutations as part of IPSS-M to help predict long-term outcomes in patients following allo-HCT [34,36]. Thus, evidence suggests that knowledge of somatic mutations can help guide decision-making surrounding cytoreductive therapy and potentially conditioning intensity in allo-HCT for MDS.

The first notable study that investigated this relationship was from Bejar et al. in 2014, who examined 125 patients with MDS that underwent allo-HCT between 2004 and 2009 [62]. They were able to identify 17 genes that were mutated in 5% or more of the patients in the cohort. Within a univariate analysis, only *TP53* mutations were associated with shorter OS (HR 3.74) and shorter PFS (HR 3.97). *TET2* and *DNMT3A* mutations were also associated with shorter OS in the adjusted multivariate analysis. Consistent with previous findings, bone marrow blasts ≥ 5% had worse OS compared to <5% (HR 1.84). They did not find different outcomes when comparing MAC versus RIC regimens. Della Porta et al. reported similar findings where mutations in *TP53* as well as *ASXL1* and *RUNX1* were independent risk factors for poor OS in patients with MDS or secondary AML (N = 401) who received allo-HCT [63]. Yoshizato et al. reported that *TP53* mutations, in combination with complex karyotype, conferred poor OS, but in contrast, *TP53* mutations alone had fairly good OS post-allo-HCT [64]. Hunter et al. added to these findings by performing serial molecular profiling on 47 patients with *TP53*-mutated MDS treated with HMA-based therapy, with 16 patients proceeding to allo-HCT [65]. In the patients who underwent allo-HCT, persistence of *TP53* mutation with a variant allele frequency (VAF) of ≥5% resulted in similarly poor outcomes compared to patients who did not receive an allo-HCT. Conversely, patients with a VAF < 5% experienced a survival benefit compared to those who remained on HMA and did not proceed to allo-HCT with a median of 25.2 versus 7.7 months. In contrast, Versluis et al. conducted a genetic analysis of the BMT CTN 1102 study of older patients with MDS who did or did not proceed to allo-HCT [66]. Through a time-dependent analysis, independent of *TP53* mutations, patients appeared to have longer OS with allo-HCT who received RIC, compared to those without HCT, with a 3-year OS of 23%. Further, 94% of patients had persistent *TP53* mutations pre-allo-HCT, with no difference in 3-year OS between those with a VAF ≥5% versus <5% (OS 22% versus 18%). Collectively, these findings demonstrate the controversy of whether allo-HCT confers benefits for patients with *TP53*-mutated MDS. To complicate matters, it is well established that *TP53*-mutated MDS is fairly chemo-resistant [40,67,68], with infrequent and short-lived responses to HMA therapy even when combined with venetoclax, making it increasingly challenging to clear *TP53* mutations before allo-HCT. Unfortunately, the negative results of the phase III ENHANCE clinical trial (NCT04313881) that failed to confirm the exciting results from phase I and II data in patients with *TP53* mutations treated with magrolimab and azacytidine emphasize the difficulty in treating this subset of MDS patients. The hope is that one of the many novel approaches [69,70] to treating this condition will be clinically useful in significantly decreasing disease burden prior to transplant.

Additional studies have examined whether conditioning intensity may impact patient outcomes following allo-HCT depending on the somatic mutational profile. One of the largest studies was by Lindsley et al., who used the CIBMTR database to examine the relationship between somatic mutations and conditioning intensity on outcomes following allo-HCT in patients with MDS (N = 1514) [71]. *TP53* mutations were present in 19% of patients, and this study confirmed the worse OS (HR 1.96) and shorter time-to-relapse (HR 2.03). *TP53* VAF and biallelic *TP53* mutations did not impact survival. In patients without *TP53* mutations, RAS-pathway mutations (*NRAS*, *KRAS*, *PTPN1*, *CBL*, *NF1*, *FIT1*, *FLT3*, and *KIT*) conferred relatively shorter time-to-relapse (HR 1.56). They looked at whether conditioning intensity could overcome the poor prognosis of these mutations. In patients with *TP53* mutations, the median OS was similar between MAC and RIC at 7.5 months and 9.2 months, respectively, with no difference in incidence of relapse. Conversely, in patients with RAS-pathway mutations, there was a higher risk of relapse in the RIC group at 42% versus 20% in those without RAS-pathway mutations, whereas differences were not seen with MAC. In the multivariate model, *TP53* mutations were found to be the most powerful independent predictor of OS following allo-HCT in patients with MDS. More intensive conditioning did not attenuate this mutation’s effect on relapse and death. As such, they suggested that escalation of conditioning intensity provides no benefit to overcome the negative effects of *TP53* mutations. With respect to RAS-pathway mutations, they suggested that MAC may provide benefits for reducing early relapse over RIC when the safety profile is acceptable. These findings are supported by Chan et al., who found that in patients with MDS/AML with TP53 persistence at the time of allo-HCT, conditioning with MAC led to inferior OS compared to RIC (HR 2.6) [72]. Similar findings have been seen with AML, where the BMT-CTN 0901 study found no influence of conditioning on the incidence of relapse or survival in *TP53*-mutated disease [73]. In contrast to these findings, Byrne et al. reported that in MDS/AML patients with *TP53* mutations, RIC/NMA conditioning intensity increased the risk of post-allo-HCT relapse (HR 2.54) [74].

In summary, the presence of somatic mutations prior to allo-HCT appears to influence long-term outcomes. The benefit of cytoreductive therapy and the intensity of cytoreduction in the context of high-risk somatic mutations in MDS have yet to be addressed directly in a clinical trial. Further, how cytoreductive therapy prior to allo-HCT versus upfront allo-HCT affects long-term outcomes depending on the presence of high-risk somatic mutations is not well known. On the other hand, somatic mutations seem useful in guiding the intensity of the conditioning regimen or at least avoiding circumstances where the increased intensity is ineffective—thus, given the lack of difference in outcomes between MAC and RIC regimens in *TP53*-mutated MDS, it seems reasonable to proceed with RIC, provided that other patient and disease factors have been taken into consideration.

## 5. Relationship Between Minimal Residual Disease and Conditioning Intensity Prior to Allogeneic Hematopoietic Stem Cell Transplant in MDS

The primary role of chemotherapy in conditioning regimens is to eliminate any residual disease present at the time of allo-HCT. Ideally, the combined effect of prior treatments to reduce disease burden and the intensity of the conditioning regimen should interact in an additive or synergistic manner. There are emerging data to suggest that the MRD status is a powerful predictor of post-transplant outcomes. This concept has been clearly demonstrated in the setting of AML, where the presence of *FLT3* or *NPM1* MRD as assessed by very sensitive assays measured prior to conditioning can predict clinical outcomes and help guide decision-making for allo-HCT in otherwise good-risk patients and the utility of post-transplant maintenance. With respect to patients with *FLT3*-mutated AML, the MORPHO trial demonstrated that patients with undetectable MRD status at the time of transplant did well with less intensive conditioning regimens, while those with detectable MRD benefited from more intensive regimens [75]. In AML, effective treatment can diminish the disease burden to exceedingly low levels of detectable transcripts. When combined with the achievement of CR, this likely indicates a total log reduction in disease burden exceeding eight orders of magnitude prior to transplantation. In contrast, patients with MDS have historically received treatment with HMAs such as azacitidine and decitabine as single agents. It is also important to note that the historical CR rate for these agents in MDS patients is approximately 15 to 20%. Consequently, significantly fewer MDS patients may proceed to transplantation with a one- to two-log reduction, compared to the more effective disease control achieved through intensive chemotherapy for AML. Furthermore, data from the VIALE A trial indicates that AML patients treated with azacitidine monotherapy who did achieve a CR exhibited a low level of MRD clearance, or ~10%, at a relatively high threshold of 10^−4^ [76]. In contrast, the experimental arm combining venetoclax and azacitidine demonstrated MRD negativity in 40% of patients achieving CR. Given that this latter combination is not formally yet approved (pending the results of the VERONA trial) or widely utilized for MDS, it can be inferred that the majority of MDS patients, even if treated, will experience a modest (if any) log reduction in disease burden prior to transplantation. Pursuing more effective treatment regimens has proven to be exceedingly challenging, as evidenced by recent failures in randomized trials for MDS [77,78]. Therefore, one might anticipate observing a conditioning regimen intensity effect akin to that demonstrated for AML, should more effective pre-transplant therapies become available.

The use of MRD status in the setting of MDS is still emerging and has not been globally applied as a biomarker. Studies suggest that pre- and post-allo-HCT MRD status may be a risk factor for poor prognosis in MDS [79,80,81]. For example, Ma et al. in 2023 compared outcomes of patients with MDS that had either MRD-negative (N = 36) or MRD-positive (N = 67) status prior to allo-HCT [82]. The 3-year disease-free survival (DFS) was 85.6% and 73.9% in the MRD-negative and MRD-positive groups, respectively. Through both a univariate and multivariate model, they found that MRD status significantly impacted relapse. These findings suggest that MRD clearance prior to allo-HCT may confer better long-term outcomes. The aforementioned Nordic study used sensitive ddPCR and was able to show a graded increase in relapse depending on the level of MRD detection (1-year RFS of 49%, 39%, and 30% using cut-off positive levels of 0.1%, 0.3%, and 0.5%, respectively) [48]. Despite these promising findings, these studies have not specifically addressed the question of whether a higher conditioning intensity can overcome the negative impact of MRD.

Two other important studies have attempted to answer this question, though. Festuccia et al. coined the term minimal identifiable disease (MID), which is analogous to MRD status, using multiparameter flow cytometry (MFC) and cytogenetics [83]. They examined the outcomes of 287 patients with MDS. Of those, 219 patients were in morphologic remission, with 53.7% having MID-positive disease. Consistent with previous findings [82], they found that MID detected by either flow cytometry or cytogenetics resulted in worse OS. The impact of conditioning intensity differed depending on MID status, where in patients with MID-positive disease, the risk of mortality and relapse was higher in those who received RIC compared to MAC. Conversely, in patients who were MID-negative, there were similar outcomes when comparing RIC to MAC regimens. As such, they concluded that the MID status should be considered when deciding on conditioning intensity, as MID-positive disease appears to benefit from higher-intensity conditioning. A similar study by Dillon et al. in a phase III BMT CTN 0901 trial randomized patients with MDS in morphologic remission to conditioning with either MAC or RIC and assessed MRD status prior to conditioning [84]. They used ultra-deep DNA sequencing for mutations in 10 gene regions previously shown to be high risk in patients with AML [85]. Of the 48 patients, 42% had MRD-detectable disease. When comparing MRD-positive to MRD-negative disease outcomes, the 3-year RFS was 34% versus 71%, and 3-year OS was 55% versus 79%, respectively. Next-generation-sequencing (NGS) served as a strong predictor of relapse at 24 months. In MRD-positive disease, those who received RIC had higher rates of relapse (3-year relapse, 75% RIC versus 17% MAC) and lower RFS (3-year RFS, 13% RIC versus 49% MAC), with a median time-to-relapse of four months. There was no difference in TRM or OS when stratifying according to NGS status and conditioning intensity. In patients with MRD-negative disease, there was no difference in clinical outcomes between the conditioning intensities. As such, they demonstrated higher relapse in patients with MRD-positive status who were treated with RIC as opposed to MAC regimens.

While MDS is a different disease entity, these aforementioned studies have findings similar to those reported for AML. For example, Hourigan et al. assessed post-allo-HCT outcomes in patients with AML depending on pre-transplant MRD status and conditioning intensity [85]. In patients with detectable mutations via NGS, the 3-year relapse risk was higher in those who received RIC as opposed to MAC at 67% versus 19%, respectively. Similarly, 3-year OS was lower in those who received RIC at 43% versus 61% in the MAC group. Conversely, in patients with MRD-negative disease, the increased relapse in the RIC arm saw an equivalent increase in TRM in the MAC arm, resulting in similar post-transplant survival outcomes. In line with the concept of a differential anti-leukemic activity of different RIC regimens, the recently published Pre-MEASURE trial in AML showed that melphalan-containing conditioning regimens resulted in outcomes more similar to MAC regimens, where patients with NGS-MRD who received melphalan-based RIC had reduced rates of relapse at three years (44%) and improved survival compared to those with NGS-MRD who received non-melphalan based conditioning (87%) [86]. The ongoing MEASURE trial (NCT05224661) will hopefully further elucidate the clinical utility of MRD testing for relapse and survival in patients with AML undergoing allo-HCT.

At present, there are no guidelines on the routine use of MRD testing in MDS; however, the International Working Group (IWG) 2023 response criteria for higher-risk MDS suggest using MRD as an exploratory end point [87]. It is becoming apparent that the use of MRD testing can identify patients who are at higher risk of relapse following allo-HCT and, hence, would benefit more from MAC as opposed to RIC regimens. In line with this, there appear to be comparable outcomes between MAC and RIC regimens in those with MRD-negative disease, suggesting that a less toxic regimen may be reasonable, especially in older or medically infirm patients. There is a future need to develop scoring tools that incorporate both MRD status and conditioning intensity to help predict patient outcomes following allo-HCT.

## 6. Role of the Graft-Versus-Tumor Effect in MDS

An essential determinant of post-transplant outcomes is establishing effective and durable immune control of residual disease under the GvT effect. As discussed, this involves optimizing (1) disease debulking via the conditioning regimen and any preceding treatment, (2) the successful organization of the anti-tumor lymphocyte response, and (3) appropriate control of this response to forestall the development of severe GvHD.

The negative result of the FIGARO study with respect to dose intensity was counterbalanced by an unexpected positive finding: the degree of T cell chimerism at 3 months emerged as the most robust predictor of subsequent relapse-free survival in MDS patients [20], with MRD post-transplant being the strongest determinant: post-transplant positive MRD only worsened outcomes in patients with mixed donor T cell chimerism. The negative prognostic value of mixed T cell chimerism has similarly been demonstrated across multiple studies at different timepoints post-HCT [88,89,90]. These findings point to the GvT immune response, rather than conditioning intensity as explicitly tested in the trial, as a key factor governing relapse risk, suggesting that a robust T cell graft can act to intercept disease relapse from MRD.

The additional antileukemic function of donor T cells beyond host T cells is attributable to alloreactivity against mismatched host human leukocyte antigens (HLAs) or minor histocompatibility antigens (miHAs), resulting in the elimination of residual malignant cells from the host [91]. Indeed, transplants from identical twin donors with perfect histocompatibility exhibit significantly higher rates of leukemia relapse [92]. Alloreactivity triggers a strong cytotoxic response in donor T cells, specifically originating from the grafted memory and naive CD8+ T cell compartment, which reconstitutes prior to its helper CD4+ counterpart within ~100 days of transplantation [93,94]. HLA mismatches likewise trigger NK cell-mediated alloreactivity via incompatibility with self-recognizing iKIR receptors [95]. In contrast, T effectors maturing from graft-derived progenitors are non-alloreactive, having undergone host tolerization in the thymus. Instead, these effectors are restricted to conventional anti-tumor cytotoxicity via targeting tumor-associated antigens, such as MAGE or WT1, or tumor neoantigens, which are scarce in myeloid malignancies given their low relative mutational burden [96,97,98].

An intriguing area for further exploration is whether it is the conditioning regimen intensity in its entirety or rather specific drugs within the regimens that influence T cell chimerism during the critical early months post-transplant, as indicated by the findings from the FIGARO trial [20]. Fludarabine is one chemotherapeutic agent commonly utilized in various conditioning regimens with active metabolites that are well-documented to have cytotoxic effects on CD4+ and CD8+ T-lymphocytes through different mechanisms [99]. Fludarabine is often based on body surface area, with significant variability in exposures being reported [100,101,102,103]. Fludarabine overexposure has been reported to be associated with reduced EFS as a function of increased NRM as well as delayed T cell reconstitution in patients with AML. The delay in immune reconstitution associated with fludarabine has been associated with an increased risk of relapse, although this has not been consistently demonstrated [99]. Higher dosing of fludarabine in MAC compared to RIC protocols could, therefore, result in a greater suppressive effect on T cell reconstitution and explain differences in relapse incidence and NRM that are seen in MDS where the reliance on GVT may be greater. Descriptive studies of post-transplant immune reconstitution suggest that MAC represses the mitogenic capacity of reconstituting T cells compared to RIC, but so far there is no clear evidence that this translates to quantitative differences in overall T cell repopulation [104,105,106]. On the other hand, GvHD prophylaxis plays a significant role in shaping immune reconstitution. For example, the use of alemtuzumab is known to confer greater prophylaxis from both acute and chronic GvHD compared to ATG, but at the cost of delayed T cell reconstitution [107]. ATG appears to primarily stall the reconstitution of CD4+ populations while only minimally hindering CD8+ T cells [108]. The clinical consequences of these distinct immunological effects were reflected in a retrospective study of the EBMT MDS registry comparing alemtuzumab vs. ATG vs. no T cell depletion (*n* = 168/646/470), where the benefit of additional GvHD protection by alemtuzumab appeared to be counteracted by an increased relapse risk (RR 2.18 vs. no T cell depletion), yielding worse survival (HR 1.38). In contrast, ATG resulted in a lower relapse risk (RR 1.35) with similar OS compared to no T cell depletion while still conferring GvHD protection, suggesting that it provides a superior trade-off between GvT and GvHD [109]. Although optimizing immune reconstitution through earlier tapering of immunosuppression conceptually could be expected to improve graft function and trials evaluating earlier cessation as early as Day 60 have demonstrated overall safety, there has not been a consistent benefit in disease-related outcomes [110]. Institutional and national protocols often specify which type of GvHD prophylaxis is to be used in combination with specific conditioning chemotherapeutic agents for distinct disease indications. Further complicating the evaluation is the combination of immunosuppressants that are utilized with the respective conditioning regimens (i.e., methotrexate and cyclosporine seen in myeloablative protocols; tacrolimus, mycophenolate mofetil, and post-transplant cyclophosphamide as in the Baltimore protocol, etc.). Therefore, given the interaction of these variables being prescribed in specific combinations, teasing out which agent in the regimen is responsible for the effects remains a challenge and requires further investigation.

Donor lymphocyte infusion (DLI) represents one approach to boost antitumor immunity after transplant. In the relapse setting, DLI achieves response rates of ~30% in AML/MDS mixed cohorts, with a 2-year OS of ~20% [111,112,113]. Although representing an improvement over salvage chemotherapy, the modest survival results suggest that GVT cannot fully control disease once frank relapse has occurred [111]. On the other hand, there is some observational evidence in MDS indicating that earlier DLI in individuals with declining chimerism and cytopenia without molecular or cytologic relapse can yield better results [114]. Based on the hypothesis that the “window of opportunity” for GvT occurs earlier in time, prophylactic approaches to DLI based on MRD status or cytogenetic risk are under active study and appear to confer significant benefit in AML, although MDS data remains limited [115,116]. However, this will be explicitly addressed in the UK IMPACT PRO-DLI trial (NCT02856464), where patients will be randomized to prophylactic DLIs if GvHD is absent, compared to “on-demand” DLIs for mixed chimerism and post-transplant MRD positivity.

The dependence of GvT on alloreactivity makes it inextricably entangled with GvHD. In AML, it has long been recognized that some graft-versus-host (GvHD) reflects a greater underlying immune activation that could forestall relapse. For instance, it has been shown that fast reconstitution of CD8+ is protective against disease relapse while increasing the risk of GvHD [20]. In the context of MDS, a large retrospective study by Konuma et al. (*n* = 3119) specifically demonstrated this relationship between GvT and risk of relapse. For patients with high-risk MDS, limited cGVHD was linked to a significant reduction in risk of subsequent disease relapse (HR 0.57), which corresponded with a decrease in overall mortality (HR 0.66). In contrast, while grade III–IV aGVHD and extensive cGVHD also lowered relapse rates, their benefits were negated by an increase in NRM. Notably, the GvT effect associated specifically with limited cGVHD contributed to improved OS in high-risk MDS [117].

Significant efforts have also been made to identify strategies to expand the window between protective GvT and detrimental GvHD by exploiting factors specific to one of the two phenomenona. One such method uses hypomethylating agents to increase the expression of minor histocompatibility antigens and tumor-associated antigens selectively on leukocytes, exposing them to T cell and NK cell killing. A descriptive study by Cruijsen et al. showed that adjunctive decitabine, particularly during transplant conditioning, led to effective induction of T cell responses against both miHAs and tumor-associated antigens in a cohort of AML and MDS patients with GvHD risk comparable to conventional conditioning [118]. This was further supported by results from a retrospective study by Zhang et al. where the addition of decitabine to MAC (Bu/Cy) significantly improved survival in high-risk MDS (70.2% vs. 51.1% at 3y) [119]. On the other hand, efforts are also investigating the specific prevention of GvHD by reducing lymphocyte trafficking to target organs. For instance, preclinical work has demonstrated that blockade of IFN-ɣ and IL-6 signaling using the JAK inhibitor baricitinib ameliorated GvHD while maintaining T cell reconstitution and GvT [120].

Overall, the evidence suggests that the effectiveness of GvT is an additional key determinant of MDS outcome. Post-transplant T-cell chimerism appears to be a good predictor of the GvT effect. However, the extent to which it influences relapse risk independently of overall host-graft chimerism remains unclear. Nevertheless, modulation of the GvT effect via selecting different T cell depletion strategies, giving DLI, or hypomethylating agents could be effective strategies for improving outcomes. The implication is that there may be an ideal profile of immune reconstitution that strikes an optimal balance between GvT and GvHD, which will need to be clarified through further study of immune reconstitution patterns, ideally via direct measurements of key effector subpopulations responsible for GvT.

## 7. Conclusions

Given the stem cell origin and heterogeneity of myelodysplastic syndromes (MDSs), transplant outcomes are primarily influenced by disease biology—most notably specific genetic alterations—and the timing of transplantation. In contrast, pretransplant treatment response appears to have limited prognostic significance. Whether this will change with the introduction of more active therapies or more sensitive tools for response assessment remains to be seen. Increasing conditioning intensity alone has not consistently improved outcomes; however, using agents with lower systemic toxicity and potentially greater selectivity for transformed stem cells has shown encouraging trends in clinical trials. Importantly, the success of transplantation also highlights its role as a form of cellular immunotherapy, with the immunologic graft-versus-MDS effect emerging as a critical determinant of long-term disease control.

## Figures and Tables

**Table 1:** Conditioning regime intensity for MDS.

Trial	Condition	Regimen	Type of Study	Patients	Outcome
Multicenter retrospective study (Martino, Blood 2006) [29]	MDS	RIC vs. standard myeloablative conditioning	Registry Analysis	Total 836 patients	OS at 3 years 45% for MAC vs. 41% for RIC, *p* = 0.8 CIR at 3 years 27% for MAC vs. 45% for RIC, *p* < 0.01
BMT CTN 0901 (Scott et al., JCO 2017) [15]	AML and MDS	RIC regimens: fludarabine with busulfan (≤8 mg/kg orally or 6.4 mg/kg intravenously; Flu/Bu2) or melphalan (≤150 mg/m^2^, Flu/Mel). MAC regimens: busulfan (16 mg/kg orally or 12.8 mg/kg intravenously) with cyclophosphamide (120 mg/kg) or fludarabine (120 to 180 mg/m^2^; Flu/Bu4) or cyclophosphamide (120 mg/kg) and total-body irradiation (12 to 14.2 Gy)	RCT	Total 272, 54 with MDS (terminated early because of benefit of MAC in total group)	OS for MDS at 18 months: 81.5% for MAC vs. 85.2% for RIC, *p* = 0.175 CIR for MDS at 18 months: 3.7% for MAC vs. 37.0% for RIC (*p* value not calculated by confidence intervals do not overlap)
RICMAC Trial (Kröger et al., JCO 2017) [17]	MDS	MAC: busulfan (16 mg/kg orally or 12.8 mg/kg intravenously) and cyclophosphamide (120 mg/kg). RIC: busulfan (8 mg/kg orally or 6.4 mg/kg intravenously) and fludarabine (150 mg/m^2^)	RCT	Total 129 with MDS (terminated early because of slow accrual)	OS at 2 years: 63.2% for MAC vs. 76.3% for RIC, *p* = 0.08 CIR at 2 years: 14.8% for MAC and 17% for RIC, *p* = 0.64
CIBMTR Analysis (Oran et al., TCT 2021) [22]	MDS	fludarabine with either total melphalan dose ≤ 150 mg/m^2^, or busulfan ≤ 7.2 mg/kg intravenously (IV)	Registry Analysis	Total 1045 patients	OS at 2 years 61% for FluBu vs. 63% for FluMel, *p* = 0.4 CIR at 2 years 47% for FluBu vs. 28% for FluMel, *p* < 0.0001
MC-FludT.14/L (Beelen et al., Lancet Haematology, [26] Beelen et al., AJH 2022 for final analysis) [30]	AML and MDS	intravenous (IV) fludarabine with either treosulfan (30 g/m^2^ IV) or busulfan (6.4 mg/kg IV)	RCT	Total 570 patients, MDS 199 patients	OS at 3 years 56.3% in FluBu and 66.8% % in FluTreo, *p* = 0.0037 CIR at 3 years 26% in FluBu and 25.9% in FluTreo, non-significant
University Leipzig analysis (Jentzsch et al., 2019) [31]	MDS and MDS/MPN	NMA vs. RIC	Single-center retrospective	Total 151 patients	No difference in OS *p* = 0.21, CIR, *p* = 0.38
EBMT analysis (Shimoni et al., BJH 2021) [27]	MDS	MAC vs. RIC vs. Treo	Registry Analysis	Total 1722 patients	OS at 5 years 50% in FluTreo and 43% in MAC and 43% in RIC; *p* = 0.03; 5 year CIR 25% FluTreo, 25% for MAC, 38% for RIC, *p* < 0.001
Figaro (Craddock et al., JCO 2020) [20]	AML/MDS	FLAMSA-Bu vs. Control (FluBu or FluMel)	RCT	Total 244 patients, MDS 78 patients	OS at 2 years 58.8% in control group and 60.9% in FLAMSA-Bu, *p* = 0.81 CIR at 2 years 29.5% in control group and 26.7% in FLAMSA-Bu, *p*= 0.81
EBMT analysis MAC vs. RIC vs. sequential (Potter et al., BMT 2024) [31]	MDS	MAC vs. RIC vs. Sequential Conditioning	Registry Analysis	Total 303 patients	OS at 3 years 62% for MAC, 46% for RIC, 52% for sequential conditioning *p* = 0.13 CIR at 3 years 18% for MAC, 25% for RIC, 22 for sequential conditioning, *p* = 0.14
PMCC Analysis (Pasic et al., 2024) [28]	MDS	Treosulfan vs. Busulfan	Single-center Retrospective Propensity Score-matched Cohort Study	Total 138 patients	OS at 2 years 66.9% for FT and 44.5% for FBT200 (*p* = 0.013) CIR at 2 years 15.6% for FT and 27.6% for FBT200 *p* = 0.22

## Abbreviations

aGvHD	acute graft-versus-host disease
AML	acute myeloid leukemia
Allo-HCT	allogeneic hematopoietic stem cell transplant
ASXL1	additional sex combs-like protein 1
ATG	anti thymocyte globulin
BM	bone marrow
BMT CTN	Blood and Marrow Transplant Clinical Trials Network
CIBMTR	Center for International Blood and Marrow Transplant Research
CBL	Casitas B-lineage lymphoma
DLI	donor lymphocyte infusion
DNMT3A	DNA methyltransferase 3A
EBMT	European Society for Blood and Marrow Transplantation
*FIT1*	fat storage-inducing transmembrane protein 1
FLAMSA	fludarabine amsacrine cytosine arabinoside
*FLT3*	FMS-related tyrosinekinase 3
Flu/Bu	fludarabine busulfan
Flu/Mel	fludarabine melphalan
cGvHD	chronic graft-versus-host disease
GVT	graft versus tumor effect
HCT	hematopoietic stem cell transplant
HCT-CI	hematopoietic stem cell transplant comorbidity index
HLA	human leukocyte antigen
HMA	hypomethylating agent
HR	hazard ratio
IFN	interferon
iKIR	inhibitory killer cell immunoglobulin-like receptor
IPSS	International Prognostic Scoring System
IPSS-M	International Prognostic Scoring System—Molecular
IPSS-R	International Prognostic Scoring System—Revised
IWG	International Working Group
JAK	Janus kinase
MAC	myeloablative conditioning
MAGE	melanoma antigen gene
MDS	myelodyplastic syndrome
MFC	multi-color flow cytometry
MID	minimal identifiable disease
miHA	minor histocompatibility antigen
MRD	measurable residual disease
*NF1*	neurofibromatosis 1
NGS-MRD	next generation sequencing measurable residual disease
NK	natural killer
*NPM1*	nucleophosmin-1
NRM	non-relapse mortality
OS	overall survival
PFS	progression free survival
RFS	relapse free survival
PTPN1	protein tyrosine phosphatase 1B
RIC	reduced intensity conditioning
RCT	randomized control trial
*RUNX*	runt-related transcription factor
*TP53*	tumor promoter 53
TRM	treatment related mortality
VAF	variant allele frequency
WT1	Wilm’s tumor 1
